# Chondroblastic osteosarcoma of the nasal cavity: an exceptional and misdiagnosed presentation

**DOI:** 10.1093/jscr/rjae409

**Published:** 2024-06-06

**Authors:** Taha Yassine Aaboudech, Hafsa EL Ouazzani, Habiba Kadiri, Hanae Benabdenbi, Naji Rguieg, Ibtissam EL Ouali, N Ech-cherif EL Kettani, Fouad Zouaidia, Nadia Cherradi

**Affiliations:** Pathology Department, Rabat Specialty Hospital, Rabat 10100, Morocco; Mohammed V University in Rabat, Rabat 10000, Morocco; Pathology Department, Rabat Specialty Hospital, Rabat 10100, Morocco; Mohammed V University in Rabat, Rabat 10000, Morocco; Pathology Department, Rabat Specialty Hospital, Rabat 10100, Morocco; Mohammed V University in Rabat, Rabat 10000, Morocco; Pathology Department, Rabat Specialty Hospital, Rabat 10100, Morocco; Mohammed V University in Rabat, Rabat 10000, Morocco; Pathology Department, Rabat Specialty Hospital, Rabat 10100, Morocco; Mohammed V University in Rabat, Rabat 10000, Morocco; Mohammed V University in Rabat, Rabat 10000, Morocco; Neuroradiology Department, Rabat Specialty Hospital, Rabat 10100, Morocco; Mohammed V University in Rabat, Rabat 10000, Morocco; Neuroradiology Department, Rabat Specialty Hospital, Rabat 10100, Morocco; Mohammed V University in Rabat, Rabat 10000, Morocco; Pathology Department, Ibn Sina Hospital, Rabat 10100, Morocco; Pathology Department, Rabat Specialty Hospital, Rabat 10100, Morocco; Mohammed V University in Rabat, Rabat 10000, Morocco

**Keywords:** chondroblastic osteosarcoma, nasal cavity, histopathology, case report

## Abstract

Chondroblastic osteosarcoma of the nasal cavity is a rare and aggressive tumor that presents diagnostic challenges due to its variable clinical and radiological features. Histological analysis is crucial for an accurate diagnosis, despite morphological similarities with chondrosarcoma. We present a case of primary craniofacial chondroblastic osteosarcoma originating from the nasal cavity in a 17-year-old adolescent. The tumor was characterized by cartilaginous lobules and spindle cell proliferation with osteoid matrix deposition. Immunohistochemical analysis supported the diagnosis. This case highlights the rarity of this particular presentation and emphasizes the significance of accurate diagnosis through histopathological evaluation to achieve optimal management.

## Introduction

Osteosarcoma (OS) arises from primitive bone-forming mesenchymal cells [1]. As the most common primary bone malignancy, it exhibits two age distribution peaks and typically occurs in the metaphyseal growth plates of long bones [1]. In the head and neck, craniofacial OS is exceptionally rare, comprising <0.5% of all malignancies in this area and constituting 6–13% of all OSs [1, 2]. Craniofacial OS most commonly arises in areas associated with mastication, such as the mandible and maxilla, with only a few cases reported in other facial bones. Consequently, the nasal cavity represents an exceptional primary site for OS [1]. The chondroblastic subtype, as defined by the fifth edition of the WHO in 2020, is characterized by the predominant presence of a chondroid matrix, often exhibiting a high degree of hyaline cartilage, intimately associated with non-chondroid elements such as osteoid or bone matrix [3]. This study presents a rare case of primary craniofacial chondroblastic OS arising in the nasal cavity. It emphasizes unique histopathological features and discusses diagnostic challenges, especially in distinguishing it from more common chondromatous lesions in this location.

## Case report

We present a case of a 17-year-old adolescent with no notable medical history who presented to the ENT (Ear, Nose, and Throat) department with nasal obstruction, unilateral epistaxis, blurry vision in the left eye, and headaches persisting since childhood.

A nasal fibroscopy revealed an ulcerative and budding lesion in the left nasal fossa, which was friable and hemorrhagic, infiltrating the middle meatus. The ophthalmological examination noted decreased visual acuity in the left eye with slight proptosis, but no alterations in ocular motility or reflexes were found.

**Figure 1:**
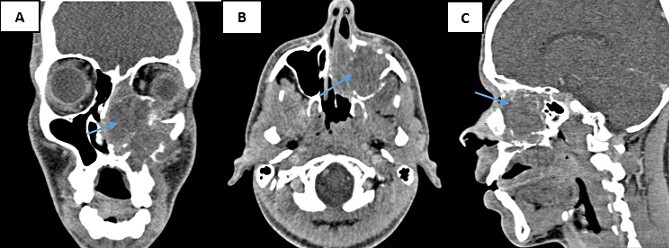
CT scans of the facial mass in coronal (**A**), axial (**B**), and sagittal (**C**) reconstructions were performed after the injection of iodinated contrast medium. The scans show a tumor (60 × 47 × 46 mm) on the left side (arrow) with hypodense tissue density. The tumor is heterogeneously enhanced after contrast injection and involves the nasal cavity and ipsilateral maxillary sinus, with aggressive infiltration of surrounding structures.

A nasal sinus computed tomography (CT) scan revealed a sizable tumoral mass measuring 60 × 47 × 46 mm, displaying heterogeneous tissue density enhancement following contrast injection. This mass originated from the nasal fossa and infiltrated several structures. Internally, the nasal septum and sphenoidal sinus; externally, the left maxillary sinus and the pterygopalatine fossa. Additionally, it involved the rectus inferior muscle, the floor, and the upper pole of the left orbit above, while causing localized lysis of the hard palate below. Posteriorly, the nasopharynx appeared intact (Fig. 1). Based on these findings, a malignant transformation of an inverted papilloma was suspected.

**Figure 2:**
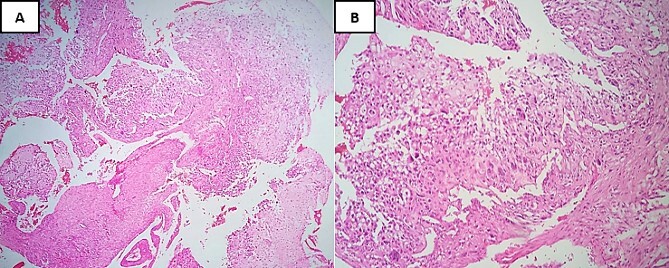
Hematoxylin and Eosin stain (H&E) under low-power magnification reveals: (**A**) areas of necrosis accompanied by a spindle cell tumor and chondroid nodules located at the periphery (×4). (**B**) Neoplastic cells typically demonstrate severe anaplasia and pleomorphism, and they are fusiform, plasmacytoid, and epithelioid merging with neoplastic cartilage (×20).

**Figure 3:**
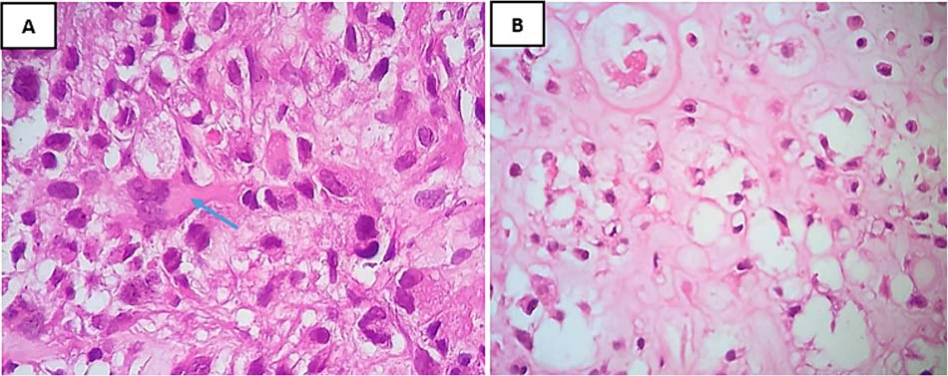
H&E staining under high-power magnification highlights: (**A**) within the spindle cell areas, tumor cells exhibit irregular hyperchromatic nuclei and brisk mitotic activity, accompanied by the production of osteoid matrix, as indicated by an arrow (×40). (**B**) The chondroid matrix contains neoplastic cells displaying cytological atypia (×40).

A biopsy for histopathological study revealed the presence of a sarcomatous tumor proliferation with significant necrosis, consisting of cartilaginous lobules as well as a proliferation of spindle cells (Fig. 2). The cartilaginous lobules exhibit high cellularity, marked by atypical, binucleated nuclei. Additionally, the spindle cells demonstrate irregular characteristics with cyto-nuclear atypia and mitotic figures, occasionally producing a tumor-specific osteoid matrix (Fig. 3).

**Figure 4:**
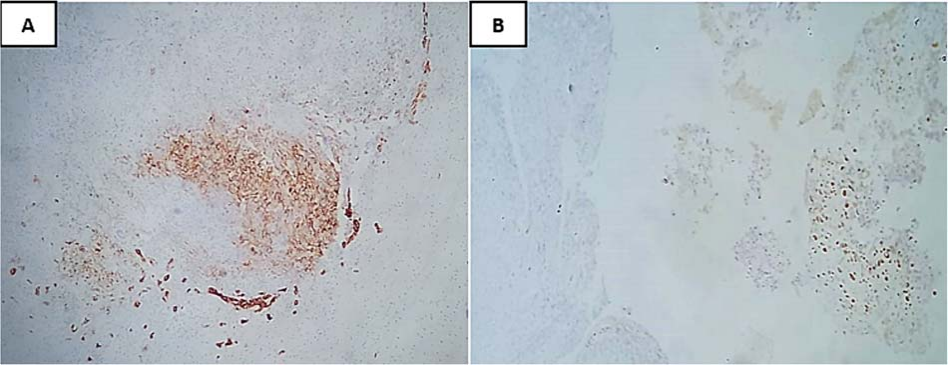
Immunohistochemical analysis showing focal staining for anti-EMA (**A**) and S100 (**B**).

The immunohistochemical analysis indicated negativity with anti-cytokeratin antibodies, anti-desmin, anti-myogenin, and anti-brachyury. In contrast, the tumor tested positive for anti-vimentin and showed focal staining for anti-EMA and S100. The Ki67 proliferation index was notably high, estimated at 90% (Fig. 4).

The patient was diagnosed with chondroblastic OS of the nasal cavity and was referred to the Oncology department for appropriate chemotherapy.

## Discussion

OS in the head and neck region make up a small fraction of all OSs, with reported incidence rates ranging from 0.5 to 8.1% [2, 4]. Typically, these tumors are diagnosed during the second or third decades of life, later than the typical onset of OSs in long bones. They often develop as secondary tumors following radiation therapy, exposure to thorium oxide, chemotherapy, inherited predispositions to OS, or from preexisting benign bone conditions such as Paget’s disease, bone infarcts, osteomyelitis, or trauma [5, 6]. Gender does not show a significant predominance [7]. The mandible and maxilla are the most common sites for craniofacial OSs [5, 8]. Sinonasal OS is an uncommon location for craniofacial OS. In Low’s review [1], which analyzed 88 patients with sinonasal OSs, the most frequent subsite affected was the maxillary sinus, followed by the ethmoid sinuses and the nasal cavity, representing 46% [1].

Clinically, chondroblastic OS, like other OSs, usually lacks specific symptoms, displaying signs commonly seen in all sinonasal tumors. The most common presenting symptoms include a facial mass or swelling, nasal obstruction, headache, epistaxis, oral mass, facial pain, and proptosis [1].

Radiological findings in chondroblastic OS are nonspecific. Panoramic radiographs may reveal either osteolytic or osteosclerotic features, sometimes both simultaneously. Cervicofacial CT scans are used to assess tumor characteristics like size, margins, calcifications, periosteal reaction, density, and local invasion. Although a ‘grass fire’ or ‘sunburst’ appearance is often seen, it lacks specificity [9]. Bone scintigraphy detects multifocal OS or metastases [10]. Magnetic resonance imaging is used to identify intramedullary, intracranial, and orbital invasion, but its limited capacity to image osseous findings is a notable drawback [11].

The precise diagnosis of craniofacial OS relies on histopathological assessment. A biopsy, ideally taken from the tumor’s center, is crucial to identify the characteristic osteoid formation typical of OS [12]. Histologically, OS is categorized into chondroblastic, osteoblastic, and fibroblastic subtypes, all of which are classified as high-grade [3].

The histopathological findings of this study have classified the tumor as a chondroblastic variant of OS. Numerous studies have indicated that this histological subtype is the most prevalent form of OS in maxillary bones, but it is the second most common subtype in sinonasal localization, after the fibroblastic subtype [1, 13]. The chondroblastic subtype is defined by a predominant component of hyaline cartilage with severe cytological atypia, but the chondroid matrix may also appear myxoid with single cells or delicate cords of cells displaying more subtle atypia, particularly in tumors arising in gnathic bones. Neoplastic cartilage usually merges with areas containing neoplastic bone, often with condensation and spindling of tumor cells at the periphery of the chondroid nodules [4]. The extensive production of chondroid matrix can complicate distinguishing between chondroblastic OS and chondrosarcoma [14]. Morphologically, the key to differentiating chondroblastic OS from chondrosarcoma lies in identifying areas where tumor cells deposit osteoid matrix. The presence of malignant osteoid matrix, along with clinical and imaging findings, is pivotal for diagnosing chondroblastic OS [15]. Currently, no immunohistochemical marker has matched the diagnostic value of this crucial morphological indicator, but some markers can aid in diagnosis. Chondroblastic OS is positive for vimentin, EMA, S100, and rarely positive for cytokeratin, whereas chondrosarcoma is positive for vimentin and S100 [16]. It is important to highlight that differentiating between these two conditions is not just an academic exercise. Chondrosarcomas are known to be resistant to radiotherapy, whereas OSs are radiosensitive. This makes accurately distinguishing between the two particularly crucial, as it can greatly influence treatment strategies and the prognosis of the lesion [13].

## Conclusion

Chondroblastic OS of the nasal cavity is an exceptional and aggressive tumor. It presents with variable clinical and radiological appearances, posing a diagnostic challenge for clinicians. Histological analysis remains pivotal for diagnosis, despite difficulties and challenges, particularly due to morphological similarities with chondrosarcoma. Accurate diagnosis is essential for optimal management.

## Data Availability

No new data were generated or analyzed in support of this research.
